# Assessing the Features of Diabetic Foot Ulcers among Individuals with Type 2 Diabetes Mellitus in Thi Qar, Iraq

**DOI:** 10.12688/f1000research.150995.2

**Published:** 2026-02-18

**Authors:** Adel Gassab Mohammed, Dheyaa Kadhim Al-Waeli, Samih Abed Odhaib, Mahmood Thamer Altemimi

**Affiliations:** 1 Thi-Qar Specialized Diabetes, Endocrine, and Metabolism Center (TDEMC), Thi-Qar Health Directorate, Nasiriyah, Thi-Qar, Iraq; 2 Thi Qar Specialized Diabetes, Endocrine and Metabolism Center, College of Medicine, University of Sumer, Nasiriyah, Thi-Qar Health Directorate, Thi Qar, Iraq

**Keywords:** diabetic foot ulcers, Type 2 Diabetes Mellitus, Thi Qar, Iraq, local studies, characteristics

## Abstract

**Background:**

This study aimed to evaluate the characteristics of diabetic foot ulcers in individuals with type 2 diabetes mellitus (T2DM) in Iraq.

**Methods:**

The study included 881 participants with T2DM and different types of foot ulcers, who attended a specialized diabetes center. Data on demographics, clinical characteristics, biochemical investigations, comorbidities, and treatment regimens were collected and analyzed.

**Results:**

The majority of the cases (96.8%) were due to T2DM, with an average age of 58 years and a mean BMI of 30 kg/m ^2^. Participants had elevated serum creatinine, blood urea, and glucose levels, with uncontrolled HbA1c levels. Comorbidities included hypertension, ischemic heart disease, diabetic neuropathy, and retinopathy. Most participants were on insulin and statins. Diabetic foot ulcers were mainly on the right foot (48%) and classified as Grade 2 in Wagner’s system. Some participants had Charcot deformity or stages of amputation.

**Conclusions:**

Random plasma glucose levels and diabetic retinopathy were significantly associated with the classification of foot ulcers. Further research is needed to explore additional variables related to T2DM and foot ulcers, emphasizing the importance of glucose control and retinopathy in ulcer classification.

## Introduction

Diabetes mellitus (DM) represents a significant global health issue, anticipated to affect 700 million people by 2045. ^1^ Diabetic foot ulcers carry a heavy burden both in economic terms and human suffering. Despite advancements in treatment and increased awareness, DFUs remain a prevalent issue among those with diabetes, especially T2DM. One of the severe consequences of DM is the formation of diabetic foot ulcers (DFUs), which can drastically diminish the quality of life for patients, lead to costly economic outcomes, and, at worst, necessitate the amputation of affected limbs. ^2^ Diabetic foot encompasses a range of foot-related health issues, from infections and ulcerations to deep tissue damage arising from the complications of diabetes itself. ^3^ An estimated 80% of non-traumatic amputations are attributed to DFUs, underscoring their severe impact. ^4^

The typical etiology of DFUs involves a combination of neuropathic, traumatic, ischemic, and infectious factors. ^5^ Globally, the prevalence of diabetic foot stands at approximately 6.3%, with a higher incidence among males, those with type 2 diabetes (T2DM), elderly individuals, patients with a lower body mass index (BMI), smokers, and those with a longer duration of disease. Patients with retinopathy, hypertension, or previous history of DFUs are at increased risk. ^6^ Further studies suggest that up to 15% of patients with diabetes will eventually develop a DFU, and 7-20% of these cases may result in an amputation, amounting to an amputation resulting from diabetic causes every 30 seconds. ^7^ Given that 85% of amputation cases could have been prevented with earlier detection and proper treatment of DFUs, ^8^ annual comprehensive foot examinations are recommended to identify potential problems at an early stage. ^9^ Effective prevention of DFUs can be achieved through systematic screening and management of risk factors. ^10^

The absence of localized studies prompted the current research, intended to appraise the characteristics and prevailing conditions of DFUs to enhance both prevention and treatment strategies for this significant health concern. The research was conducted at the Thi-Qar Specialized Diabetes Endocrine and Metabolism Center (TDEMC) and focused on evaluating the specificities of DFUs in patients with T2DM in the southern region of Iraq.

## Methods

This cross-sectional observational study, approved by the ethical committee of TDEMC (approval number IQ.TDEMC.REG.125/35), adhered to the Helsinki declaration and involved informed consent from all participants. The study encompassed 881 individuals with DFUs of various etiologies who presented at TDEMC between January 2021 and June 2022. According to the requirements of the EQUATOR (Enhancing the QUAlity and Transparency Of health Research) protocol guidelines, a cross-sectional study follow the STROBE (STrengthening the Reporting of OBservational studies in Epidemiology) guidelines.

### Study design

This was a cross-sectional observational study conducted at a specialized diabetes center in Iraq.

### Participants

A total of 881 individuals with type 2 diabetes mellitus and different types of foot ulcers were included in the study. Participants were selected based on their attendance at the diabetes center and the presence of foot ulcers.

### Data collection

Data on demographics (age, gender), clinical characteristics (BMI, blood pressure), biochemical investigations (serum creatinine, blood urea, glucose levels, HbA1c), comorbidities (hypertension, ischemic heart disease, diabetic neuropathy, retinopathy), and treatment regimens (insulin, statins) were collected from medical records and participant interviews. Chi-square tests were performed to analyze associations between clinical factors and WG classification. The p-values reported represent overall associations for each variable across all Wagner grades.

### Data analysis

Descriptive statistics were used to summarize the characteristics of the study population, including mean age, BMI, and biochemical parameters. Associations between random plasma glucose levels, diabetic retinopathy, and the classification of foot ulcers were analyzed using appropriate statistical tests.

### Ethical considerations

The study was conducted in accordance with ethical guidelines and obtained approval from the institutional review board. Informed consent was obtained from all participants before data collection.

### Limitations

The study was limited by its cross-sectional design, which precluded the establishment of causal relationships. Additionally, the study was conducted at a single center, which may limit the generalizability of the findings.

### Future research

Further research is needed to explore additional variables related to type 2 diabetes mellitus and foot ulcers, with a focus on the importance of glucose control and retinopathy in ulcer classification.

DFU properties, such as location and severity, were quantified using Wagner’s grading system (WG), which categorizes ulcers as grade 1 (superficial), grade 2 (deep), grade 3 (abscessed deep ulcer with bone involvement), grade 4 (localized gangrene), and grade 5 (extensive gangrene). The aim was to analyze the association between various clinical factors and the WG classification of DFUs.

## Results

The study cohort featured a substantial majority of T2DM cases at 96.8% (n=853) with a male predominance of 59% (n=520). The mean age was 58 years, and the cohort typically displayed characteristics of being overweight or obese, with a mean BMI of 30.00 kg/m ^2^. The average duration of diabetes among the participants was 14.0 years as illustrated in Table 1.

**Table 1.** General characteristics of the enrolled individuals with DFUs.

Variable	N (%) / Mean ± SD
A. Demographic Characteristics	
Gender: Male	520 (59.0)
Gender: Female	361 (41.0)
Age (years), Mean ± SD	58 ± 11
Age range	19–100
Age group: <40 years	45 (5.1)
Age group: 40–<60 years	436 (49.5)
Age group: ≥60 years	400 (45.4)
Marital status: Single	27 (3.1)
Marital status: Married	765 (86.8)
Marital status: Divorced	8 (0.9)
Marital status: Widowed	81 (9.2)
B. Diabetes-Related Characteristics	
Type of diabetes: Type 1 DM	28 (3.2)
Type of diabetes: Type 2 DM	853 (96.8)
Duration of diabetes (years), Mean ± SD	13.54 ± 6.98
Duration of diabetes (years), Median ± SE	12.0 ± 0.24
Duration category: ≤12 years	467 (53.0)
Duration category: >12 years	414 (47.0)
Insulin use	316 (35.9)
Family history of diabetes: Negative	414 (47.0)
Family history of diabetes: Positive	324 (36.8)
Family history of diabetes: Unknown	143 (16.2)
C. Anthropometric Measures	
Weight (kg), Mean ± SD	80.48 ± 16.11
Weight range (kg)	38–162
BMI (kg/m ^2^), Mean ± SD	30.00 ± 5.82
BMI range (kg/m ^2^)	15.03–72.00
BMI category: Underweight (<18.5)	12 (1.4)
BMI category: Normal (18.5–24.9)	149 (16.9)
BMI category: Overweight (25–29.9)	311 (35.3)
BMI category: Obesity class I (30–34.9)	261 (29.6)
BMI category: Obesity class II (35–39.9)	102 (11.6)
BMI category: Obesity class III (≥40)	46 (5.2)
D. Lifestyle and Socioeconomic Factors	
Smoking status: Smoker	137 (15.6)
Smoking status: Ex-smoker	84 (9.5)
Smoking status: Non-smoker	660 (74.9)
Occupation: Employee	109 (12.4)
Occupation: Free	201 (22.8)
Occupation: Housewife	331 (37.6)
Occupation: Retired	238 (27.0)
Occupation: Student	2 (0.2)
Educational level: Non/illiterate	201 (22.8)
Educational level: Primary school	109 (12.4)
Educational level: Secondary school	2 (0.2)
Educational level: High school	238 (27.0)
Educational level: Higher education	331 (37.6)
E. Cardiovascular and Cerebrovascular Comorbidities	
Hypertension	223 (25.3)
Ischemic heart disease (IHD)	52 (5.9)
Heart failure (HF)	5 (0.6)
Cerebrovascular accident (CVA)	18 (2.0)
Statin use	589 (66.9)
F. Microvascular and Neurological Complications	
Neuropathy	229 (26.0)
Retinopathy	167 (19.0)
Blindness	8 (0.9)
Erectile dysfunction	10 (1.1)
G. Foot-Related and Musculoskeletal Complications	
Amputation	43 (4.9)
Osteomyelitis	48 (5.4)
Charcot deformity	18 (2.0)
Wheelchair dependency	14 (1.6)

Biochemical assessments shown in Table 2 yielded a mean serum creatinine of 0.92 mg/dL, blood urea of 33.71 mg/dL, and estimated Glomerular Filtration Rate (GFR) of 88.38 mL/min/1.73m ^2^. The fasting and random plasma glucose averages were 168.3 mg/dL and 289.3 mg/dL, respectively. Notably, poor glycemic control was observed in 72.3% of participants with a mean HbA1c level of 10.26%. The lipid profile revealed mean serum cholesterol and triglyceride levels of 186.2 mg/dL and 209.78 mg/dL, respectively.

**Table 2.** Biochemical parameters of the participants.

Biochemical parameters	Description	Values
**Creatinine**	**Mean ± SD**	0.92 ± 0.35
	**Range**	0.60 – 5.30
**Blood Urea**	**33.71 ± 14.7**	10 – 125
**GFR mL/min/1.73m** ^**2**^	**Mean ± SD**	88.38 ± 19.45
	**Range**	12-130
	**G1≥90**	490 (55.6)
	**G2 (60-90)**	319 (36.2)
	**G3 (30-59)**	61 (6.9)
	**G4 (15-29)**	9 (1.0)
	**G5 (≤15)**	2 (0.2)
**FBS mg/dl**	**Mean ± SD**	168.3 ± 77.5
	**Range**	32-500
**RBS mg/dl**	**Mean ± SD**	289.3 ± 110.1
	**Range**	70-700
**HbA1c**	**Mean ± SD**	10.26 ± 2.0
	**Range**	5.8 – 17.0
	**Controlled ≤7**	47 (5.3)
	**Borderline Control 7-9**	197 (22.4)
	**Uncontrolled > 9**	637 (72.3)
**Serum Cholesterol mg/dl**	**Mean ± SD**	186.2 ± 54.26
	**Range**	77 – 360
	**<200**	538 (61.1)
	**200-239**	162 (18.4)
	**>240**	181 (20.5)
**Serum Triglycerides mg/dl**	**Mean ± SD**	209.78 ± 114.46
	**Range**	30-722
	**<150 Normal**	311 (35.3)
	**150-199 Borderline**	177 (20.1)
	**200-499 High**	363 (41.2)
	**>500 Very High**	30 (3.4)

Abbreviations: DM: diabetes mellitus, BMI: body mass index, RBS: random blood sugar, FBS: fasting blood sugar, CVA: cerebrovascular accident, IHD: ischemic heart disease, HbA1c: glycosylated hemoglobin

**Figure 1.**
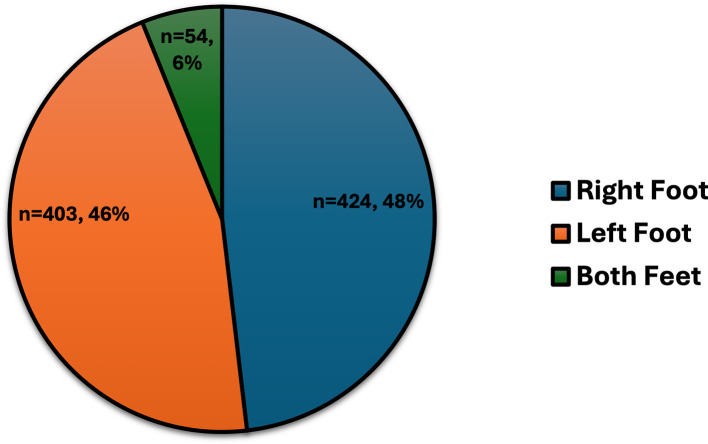
The sites of DFUs for 881 individuals with T2DM.

**Figure 2.**
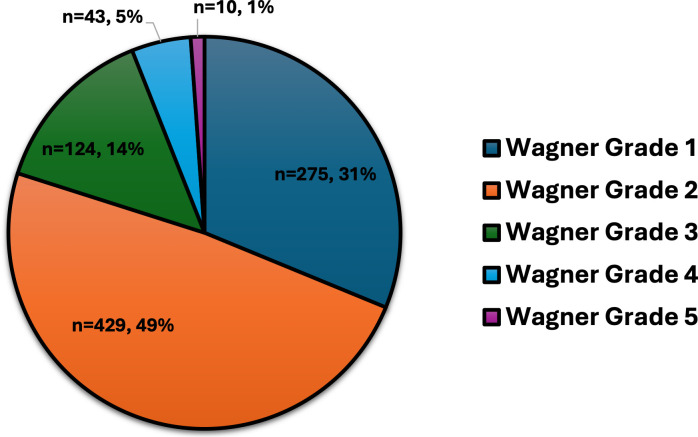
Wagner grading of DFUs for 881 individuals with DM.

DFU characteristics included ulcers predominantly on the right foot (48%), left foot (46%), or both feet (6%) ( Figure 1). According to Wagner’s grading, most ulcers were categorized as grade 1 (31%) and grade 2 (49%), with fewer instances of grades 3 through 5, which include more severe complications like deep infections, gangrene, and bone involvement ( Figure 2).

The study found statistically significant relationships for Wagner's DFU classification only with random plasma glucose levels and the presence of diabetic retinopathy as in Table 3. Other factors such as age, BMI, smoking status, family history, duration of diabetes, lipid profile, and presence of comorbid conditions like hypertension, ischemic heart disease, heart failure, and stroke showed no significant associations with Wagner's classification severity.

**Table 3.** The relationship between Wagner grading of Diabetic Foot Ulcers and different demographic, clinical, and biochemical characteristics for 881 individuals with diabetes.

Variables (Category)	Variables (Subcategory)	WG1 (n=275)	WG2 (n=429)	WG3 (n=124)	WG4 (n=43)	WG5 (n=10)	P value
**BMI**	Underweight < 18.5 (n=12)	4 (33.3%)	6 (50.0%)	2 (16.7%)	0 (0.0%)	0 (0.0%)	0.535
	**Normal 18.5-24.9 (n=149)**	38 (25.5%)	76 (51.0%)	27 (18.1%)	7 (4.7%)	1 (0.7%)	
	**Overweight 25-29.9 (n=311)**	105 (33.8%)	148 (47.6%)	35 (11.3%)	19 (6.1%)	4 (1.3%)	
	**Obesity Class 1 (30-34.9) (n=261)**	76 (29.1%)	129 (49.4%)	42 (16.1%)	12 (4.6%)	2 (0.8%)	
	**Obesity Class 2 (35-39.9) (n=102)**	40 (39.2%)	48 (47.1%)	10 (9.8%)	3 (2.9%)	1 (1.0%)	
	**Obesity Class 3 ≥ 40 (n=46)**	12 (26.1%)	22 (47.8%)	8 (17.4%)	2 (4.3%)	2 (4.3%)	
**GFR**	G1≥90 (n=490)	152 (31.0%)	240 (49.0%)	69 (14.1%)	23 (4.7%)	6 (1.2%)	0.362
	**G2=(60-90) (n=319)**	107 (33.5%)	156 (48.9%)	36 (11.3%)	16 (5.0%)	4 (1.3%)	
	**G3=(30-59) (n=61)**	14 (23.0%)	27 (44.3%)	16 (26.2%)	4 (6.6%)	0 (0.0%)	
	**G4=(15-29) (n=9)**	1 (11.1%)	6 (66.7%)	2 (22.2%)	0 (0.0%)	0 (0.0%)	
	**G5≤15 (n=2)**	1 (50.0%)	0 (0.0%)	1 (50.0%)	0 (0.0%)	0 (0.0%)	
**Smoking**	Ex-Smoker (n=84)	23 (27.4%)	39 (46.4%)	16 (19.0%)	5 (6.0%)	1 (1.2%)	0.265
	**Non-Smoker (n=660)**	219 (33.2%)	313 (47.4%)	92 (13.9%)	28 (4.2%)	8 (1.2%)	
	**Smoker (n=137)**	33 (24.1%)	77 (56.2%)	16 (11.7%)	10 (7.3%)	1 (0.7%)	
**Family History of DM**	Negative History (n=414)	128 (30.9%)	215 (51.9%)	52 (12.6%)	14 (3.4%)	5 (1.2%)	0.174
	**Positive History (n=324)**	109 (33.6%)	149 (46.0%)	46 (14.2%)	17 (5.2%)	3 (0.9%)	
	**Unknown (n=143)**	38 (26.6%)	65 (45.5%)	26 (18.2%)	12 (8.4%)	2 (1.4%)	
**Age Groups**	<40 yrs (n=45)	17 (37.8%)	15 (33.3%)	9 (20.0%)	4 (8.9%)	0 (0.0%)	0.075
	**40 to <60 (n=436)**	152 (34.9%)	203 (46.6%)	55 (12.6%)	22 (5.0%)	4 (0.9%)	
	**≥60 (n=400)**	106 (26.5%)	211 (52.8%)	60 (15.0%)	17 (4.3%)	6 (1.5%)	
**Duration Categories**	≤12 yrs (n=467)	145 (31.0%)	221 (47.3%)	67 (14.3%)	28 (6.0%)	6 (1.3%)	0.529
	**>12 yrs (n=414)**	130 (31.4%)	208 (50.2%)	57 (13.8%)	15 (3.6%)	4 (1.0%)	
**FBS Groups**	< 140 mg/dl (n=418)	134 (32.1%)	191 (45.7%)	67 (16.0%)	22 (5.3%)	4 (1.0%)	0.338
	**140-200 mg/dl (n=243)**	74 (30.5%)	123 (50.6%)	27 (11.1%)	14 (5.8%)	5 (2.1%)	
	**>200 mg/dl (n=220)**	67 (30.5%)	115 (52.3%)	30 (13.6%)	7 (3.2%)	1 (0.5%)	
**RBS Groups**	< 140 mg/dl (n=61)	12 (19.7%)	25 (41.0%)	19 (31.1%)	4 (6.6%)	1 (1.6%)	0.001
	**140-200 mg/dl (n=137)**	44 (32.1%)	59 (43.1%)	23 (16.8%)	7 (5.1%)	4 (2.9%)	
	**>200 mg/dl (n=683)**	219 (32.1%)	345 (50.5%)	82 (12.0%)	32 (4.7%)	5 (0.7%)	
**A1C Categories**	Controlled ≤7 (n=47)	12 (25.5%)	21 (44.7%)	8 (17.0%)	6 (12.8%)	0 (0.0%)	0.089
	**Borderline Control 7-9 (n=197)**	55 (27.9%)	98 (49.7%)	31 (15.7%)	8 (4.1%)	5 (2.5%)	
	**Uncontrolled > 9 (n=637)**	208 (32.7%)	310 (48.7%)	85 (13.3%)	29 (4.6%)	5 (0.8%)	
**Cholesterol Level**	<200 mg/dl (n=538)	159 (29.6%)	266 (49.4%)	81 (15.1%)	26 (4.8%)	6 (1.1%)	0.833

## Discussion

The ever-rising costs and occurrence rates of diabetes have contributed to DFUs being a leading cause of DM-related hospitalizations. ^11^ DFUs serve as strong indicators for increased mortality rates in diabetic patients due to cardiovascular or other diabetes-related complications. ^12^ Consistent with other research, the current study found a higher male prevalence, which can be partially attributed to occupational differences between genders. ^6^ However, some studies exhibit a female majority, possibly due to disparities in health care access. ^13^

Our research identified a significant association between DFUs and both random blood sugar levels and diabetic retinopathy (DRP), congruent with findings from other studies that associated DFUs with peripheral vascular disease (PVD) and neuropathy. ^14^ A similar correlation was highlighted in an Indonesian study regarding the significance of random blood sugar in the context of DFUs. ^15^

While the current study did not observe a significant association between HbA1c levels and DFU severity, the high percentage of poor glycemic control among participants indicates the impact of diabetes management on the genesis of DFUs. This is supported by other studies that link HbA1c with the severity of Wagner grading. ^16^ Contrary to some research confirming the impact of diabetes duration on DFU development, ^16^ our study and a Malaysian report found no significant correlation, potentially due to different methodologies and duration benchmarks. ^17^

Our findings regarding renal function (eGFR) showed no significant correlation with DFU severity, which contrasts with a Taiwanese study that confirmed such a correlation, especially for severe ulcers and lower limb amputations. ^18^ The difference may be due to our center treating CKD patients externally in nephrology wards, which could have influenced the recorded data.

Most of our study’s patients presented with grade 2 and grade 1 DFUs, reflecting trends observed in Iranian studies. This similarity suggests comparable healthcare practices and societal structures between the two neighboring countries. ^13^ Other research from Korea exhibited variations, which could be due to differences in treatment approaches, wound care protocols, and multidisciplinary teams. ^19^

The substantial association of DFUs with diabetic retinopathy was also noted in studies from Ethiopia and Brazil. ^20^^,^^21^ This link may be attributed to the impaired self-care abilities in those with DRP, leading to DFU development. ^22^ Moreover, DRP frequently co-occurs with peripheral neuropathy, a well-recognized DFU risk factor. ^23^ These findings suggest that ophthalmic evaluation could be beneficial for patients with severe DFU to detect potential complications at an early stage.

Despite no significant statistical correlation between BMI and DFU severity, the majority of our patients were overweight or obese, which aligns with findings from African studies. ^20^^,^^24^ Obesity is known to exacerbate atherosclerosis, a significant contributor to PVD, which plays a central role in the causation of DFUs.

Age was not significantly associated with DFU severity in our study; however, a large portion of our patients were above 60 years old, highlighting age as a factor in the development of diabetic complications, particularly atherosclerotic and neuropathic ones. This is echoed in studies from Saudi Arabia, India, and Thailand, ^25^^–^^27^ while an Ethiopian study found no significant correlation likely due to its inclusion of younger, newly diagnosed patients. ^20^

The absence of a statistically significant correlation between DFU prevalence and family history of diabetes in our study contrasts with a Chinese study that confirmed such a link. ^28^ This variance could stem from differences in populations, inadequate data collection, and the inability of many patients to recall family medical histories.

More than half of our patients had an FBS below 140 mg/dL, with no significant correlation between FBS and Wagner DFU grade, which may be due to our patients visiting the center in a fasting state and receiving regular FBS monitoring. This contradicts findings from an Indian study that noted a significant correlation. ^29^

Our findings showed no significant correlation between DFU severity and smoking, with most patients being nonsmokers, likely a result of ongoing education efforts about smoking's risks at our center. Other studies have also reported no significant association between smoking and DFU severity. ^14^^,^^30^

There was no significant correlation between DFU severity and dyslipidemia in our study, whereas an Egyptian study found a significant link. These differing findings may be due to variations in patient populations and management strategies, as most of our patients were routinely treated with statins. ^31^

### Limitations

The study is limited by its single-center, cross-sectional design. This means causal inference is not possible from it. Nor will generalizability be very wide. Moreover, it can even be said that certain clinical variables are incompletely documented. The p-values reported represent overall associations and do not specify which particular categories within each variable are driving the observed relationships. Future studies should employ post-hoc analyses or regression modeling to identify specific risk factors at the category level. These include treatment types, ulcer characteristics, peripheral pulse status and ankle–brachial index measurements.

## Conclusion

Diabetic foot ulcers carry a heavy burden both in economic terms and human suffering. Despite advancements in treatment and increased awareness, DFUs remain a prevalent issue among those with diabetes, especially T2DM. The current research provides a much-needed local perspective on the characteristics of DFUs in Thi Qar, Iraq, highlighting the clinical and biochemical variables associated with these complications.

Our research uniquely focuses on the T2DM population within a specific geographical location, which is essential for fostering strategies tailored to the local community. While random blood glucose and the presence of diabetic retinopathy had significant associations with the severity of DFUs according to Wagner’s classification, other variables such as age, BMI, and the duration of diabetes did not demonstrate a meaningful correlation. This suggests the need for more comprehensive research involving larger, diverse populations and multiple centers to draw conclusions that are more widely applicable.

The data garnered from this study contribute to the understanding of DFU prevalence and highlight the need for strict glycemic control, management of diabetes complications, and targeted interventions to prevent DFU development and progression. With concerted efforts from healthcare providers, patients, and policymakers, it is hoped that the incidence of DFUs in Iraq and similar communities can be diminished, thereby reducing the overall burden of diabetes.

## Ethics and consent

This cross-sectional observational study, approved by the ethical committee of TDEMC by the (approval number IQ.TDEMC.REG.125/35 on the 10th of December 2020), adhered to the Helsinki declaration and involved written informed consent from all participants.

## Data Availability

Zenodo: Assessing the features of diabetic foot ulcers among individuals with type 2 diabetes mellitus in Thi Qar, Iraq, https://zenodo.org/doi/10.5281/zenodo.11187872. ^32^ Data are available under the terms of the Creative Commons Attribution 4.0 International license (CC-BY 4.0).
