# Supplementary material for: Assessing the Features of Diabetic Foot Ulcers among Individuals with Type 2 Diabetes Mellitus in Thi Qar, Iraq
Source: F1000Res. 2026 Mar 26;13:539. Originally published 2024 May 28. [Version 3] doi: 10.12688/f1000research.150995.3 (PMC13100608; doi:10.12688/f1000research.150995.3)
Supplement: Peer review history

# Reviewer report: Reviewer response for version 3

**DOI:** 10.5256/f1000research.197507.r299103

**Published:** 2026-04-30

**Authors:** Marlon Yovera-Aldana

**Affiliations:** 1 Grupo de Investigación de Neurociencias, Efectividadad Clínica y Salud Pública, Universidad Cientifica del Sur, Lima, Lima, Peru

While the manuscript provides valuable descriptive data on diabetic foot ulcers (DFU) in a setting with limited published evidence, there are  conceptual and methodological concerns that  limit the interpretability and validity of the findings. Overall, the study has potential value; however,  revisions are required to ensure conceptual clarity, methodological rigor, and appropriate interpretation.

 At a general level, there is a clear misalignment between the stated objective, the analytical approach, and the conclusions. The study is presented as descriptive, yet the analysis attempts to identify “independent predictors” and the conclusions adopt a quasi-causal interpretation. Given the cross-sectional design, the manuscript should consistently frame the findings as associations rather than predictors, and avoid causal language. In its current form, the analytical framework resembles that of an explanatory study without the temporal structure required to support such claims. Additionally, because the study was conducted in a single specialized referral center, there is a high likelihood of referral bias toward more severe or complex cases, which limits generalizability and should be explicitly acknowledged.

 In the introduction, although the burden of DFU is well described, the manuscript does not clearly justify the need to characterize this population specifically in Thi Qar, Iraq. The authors should better contextualize the study within the local healthcare system, including access to primary care, referral pathways, insurance coverage, and potential delays in care. The rationale for focusing on factors associated with Wagner classification is also not sufficiently developed, particularly when more clinically meaningful outcomes—such as amputation, infection severity, hospitalization, or healing—are available. Furthermore, the choice of Wagner classification should be justified, acknowledging that it primarily captures ulcer depth and only partially reflects ischemia and infection, both of which are central to DFU severity.

 In the methods section, several issues affect reproducibility and internal validity. Although the study is described as cross-sectional, there are inconsistencies throughout the manuscript that suggest confusion with a cohort design, which should be corrected. The definition of “active DFU” is not clearly specified (e.g., duration, infection status, or clinical criteria), limiting reproducibility. There is also a major inconsistency in the study population: type 1 diabetes is listed as an exclusion criterion, yet 3.2% of participants are reported as having type 1 diabetes, while subsequent analyses appear restricted to type 2 diabetes. This discrepancy requires clarification.

 The temporal framework of variables is also unclear. It is not specified whether complications and comorbidities were assessed at admission, at discharge, or based on prior history. This is particularly relevant in a cross-sectional study, where lack of temporal clarity can lead to misinterpretation of associations. Several clinical variables are insufficiently defined. For example, peripheral neuropathy is described using multiple tests, but it is unclear whether these were applied individually or in combination, and what criteria defined a positive diagnosis. Retinopathy is treated as a binary variable without grading severity, and smoking status relies on self-report, introducing potential misclassification.

 There is also heterogeneity in data sources, as some variables appear to be directly measured while others are extracted from medical records, which introduces differential measurement bias. Importantly, key DFU-related variables are not included, such as peripheral arterial disease, ischemia severity, infection severity, ulcer duration, prior treatment, and wound microbiology. The absence of these variables significantly limits the clinical interpretability of the findings. The method used to estimate eGFR is not specified (e.g., CKD-EPI or MDRD), and there is no description of how missing data were handled. Additionally, no justification for the sample size is provided, nor is it clarified whether the sample corresponds to consecutive patients.

 The statistical analysis raises several concerns. The selection of variables included in regression models appears data-driven rather than based on a conceptual framework, increasing the risk of spurious associations. Given the number of variables included relative to the number of severe cases, there is a potential risk of overfitting. It is not reported whether collinearity or interaction effects were assessed. Continuous variables are included without evaluation of linearity assumptions or functional form, which may bias estimates. For ordinal logistic regression, key assumptions such as proportional odds are not reported.

 In the results section, the inconsistency regarding diabetes type persists and should be resolved. The timing of complications remains unclear, and some variables presented in tables are not described in the methods. The use of chi-square tests is questionable, as several cells contain very small or zero counts; it is not reported whether expected frequencies were assessed, and in such cases, Fisher’s exact test would be more appropriate. Additionally, the distribution of Wagner grades is highly unbalanced, with very few cases in higher categories, which may compromise the validity of both chi-square tests and ordinal regression models.

 The regression results are difficult to interpret due to non-standard coding of binary variables (presence = 1, absence = 2), leading to odds ratios below 1 for variables that would typically be expected to increase severity. While this is later explained, it is not the standard approach in medical literature and may confuse readers. Reanalysis using conventional coding is strongly recommended.

 Furthermore, the manuscript relies on odds ratios (OR) to quantify associations in a cross-sectional context where the outcome (e.g., severe DFU) is not rare. In such scenarios, ORs tend to overestimate the magnitude of association compared to prevalence ratios (PR), particularly when the outcome prevalence exceeds 10–20%. Given that approximately 20% of participants fall into the severe category, the use of ORs may lead to inflated interpretations of effect size. The authors should justify this choice and consider alternative approaches more appropriate for cross-sectional data, such as Poisson regression with robust variance or log-binomial models to estimate prevalence ratios. At minimum, this limitation should be acknowledged and discussed. In addition, crude (unadjusted) estimates are not presented, making it difficult to assess how associations change after adjustment, and the manuscript emphasizes p-values over effect sizes, limiting interpretability.

 In the discussion, several interpretations extend beyond what can be supported by the study design. Multiple mechanistic explanations are proposed, but these should be presented more cautiously. There is also a risk of reverse causality, particularly for variables such as random glucose, which may be elevated as a consequence rather than a cause of more severe ulcers. Some interpretations are tautological, such as linking microvascular complications to DFU severity without providing additional clinical insight. Retinopathy is discussed as if it were uniformly severe, despite being analyzed as a binary variable. The association between higher glucose levels and greater severity is expected and should be framed accordingly, rather than presented as a novel finding.

 Importantly, the discussion would benefit from a more explicit interpretation of the findings within the Iraqi healthcare context. While the manuscript justifies the study based on the lack of local data, it does not adequately relate the results to contextual factors such as access to primary care, referral pathways, healthcare infrastructure, or delays in presentation. For example, the predominance of intermediate Wagner grades and the high proportion of poor glycemic control may reflect systemic barriers to early detection and management rather than purely biological associations. Similarly, conducting the study in a specialized referral center likely concentrates more complex cases, which should be considered when interpreting the distribution of severity and associated factors. Incorporating these contextual elements would substantially strengthen the relevance, external validity, and interpretability of the findings.

 The absence of key determinants such as ischemia and infection is not adequately addressed in the discussion. The limitations section should be expanded to include residual confounding, measurement bias due to reliance on medical records, referral bias, lack of temporal clarity, and omission of key clinical variables. Additionally, the “Limitations” and “Future research” sections are currently placed within Methods and should be moved to the Discussion.

 The conclusions overstate the findings by referring to independent predictors and implying clinical applicability. Given the study design and limitations, the conclusions should be more cautious, avoid presenting specific numerical results, and clearly frame the study as hypothesis-generating rather than practice-changing.

 The manuscript would benefit from including a participant flow diagram detailing the number of patients assessed, excluded (including due to incomplete data), and included in the final analysis, in line with reporting standards.

Is the work clearly and accurately presented and does it cite the current literature?

Partly

If applicable, is the statistical analysis and its interpretation appropriate?

Partly

Are all the source data underlying the results available to ensure full reproducibility?

Yes

Is the study design appropriate and is the work technically sound?

Partly

Are the conclusions drawn adequately supported by the results?

Partly

Are sufficient details of methods and analysis provided to allow replication by others?

Partly

Reviewer Expertise:

Endocrinology; Diabetes mellitus and its complications (including diabetic foot); Clinical epidemiology; Observational study design; Biostatistics

I confirm that I have read this submission and believe that I have an appropriate level of expertise to confirm that it is of an acceptable scientific standard, however I have significant reservations, as outlined above.

# Reviewer report: Reviewer response for version 3

**DOI:** 10.5256/f1000research.197507.r470975

**Published:** 2026-04-21

**Authors:** Haeril Amir

**Affiliations:** 1 Universitas Muslim Indonesia, Makassar, Indonesia

revised manuscript is good, point by point has been updated by the authors. This is approved.

Is the work clearly and accurately presented and does it cite the current literature?

Partly

If applicable, is the statistical analysis and its interpretation appropriate?

Partly

Are all the source data underlying the results available to ensure full reproducibility?

Yes

Is the study design appropriate and is the work technically sound?

Partly

Are the conclusions drawn adequately supported by the results?

Partly

Are sufficient details of methods and analysis provided to allow replication by others?

Partly

Reviewer Expertise:

nursing management

I confirm that I have read this submission and believe that I have an appropriate level of expertise to confirm that it is of an acceptable scientific standard.

# Reviewer report: Reviewer response for version 2

**DOI:** 10.5256/f1000research.194701.r460249

**Published:** 2026-03-06

**Authors:** Haeril Amir

**Affiliations:** 1 Universitas Muslim Indonesia, Makassar, Indonesia

Summary of the Article

 This manuscript presents a cross-sectional observational study evaluating the clinical and biochemical characteristics of diabetic foot ulcers (DFU) among patients with type 2 diabetes mellitus (T2DM) in Thi Qar, Iraq. The study included 881 participants attending a specialized diabetes center between January 2021 and June 2022. The authors collected demographic data, clinical variables, biochemical markers, comorbidities, and treatment information. DFU severity was assessed using the Wagner grading system.

 The findings indicate that most participants were male, with a mean age of 58 years and an average BMI of 30 kg/m². Poor glycemic control was common among participants, with a mean HbA1c of 10.26%. The majority of ulcers were classified as Wagner grade 1 or 2. Statistical analysis showed that random plasma glucose and diabetic retinopathy were significantly associated with Wagner classification, while other variables such as age, BMI, duration of diabetes, lipid profile, and comorbidities were not significantly associated with DFU severity.

 The manuscript provides useful descriptive information regarding DFU characteristics in a local Iraqi population. The relatively large sample size and use of a standardized ulcer grading system represent strengths of the study. However, several methodological and analytical limitations reduce the strength of the conclusions and should be addressed to improve the scientific quality of the manuscript.

 Major Issues That Must Be Addressed

 These points should be addressed to strengthen the scientific validity of the manuscript.

 1. Insufficient Description of the Study Population

 The manuscript does not clearly describe the inclusion and exclusion criteria used to select participants. It is also unclear whether all patients with T2DM attending the center were screened or whether only those already diagnosed with DFU were included.

 The authors should clearly describe:

- inclusion criteria
- exclusion criteria
- sampling method (e.g., consecutive sampling, convenience sampling)
- whether patients were newly diagnosed or previously treated.

This information is essential to assess the representativeness of the sample and potential selection bias.

 2. Limited Statistical Analysis

 The statistical analysis is restricted to descriptive statistics and Chi-square tests. While this approach can identify simple associations, it is insufficient to determine independent predictors of DFU severity.

 The authors should consider performing **multivariate regression analysis (e.g., logistic regression or ordinal regression)** to identify independent factors associated with Wagner classification. This would strengthen the scientific contribution of the study and reduce the influence of confounding variables.

 3. Insufficient Description of Clinical Variables

 Several important clinical variables are mentioned but not clearly defined, including:

- diabetic neuropathy
- diabetic retinopathy
- ischemic heart disease
- smoking status

The manuscript should specify how these variables were diagnosed or measured (clinical assessment, laboratory results, imaging, or medical records).

 4. Interpretation of Results

 The discussion section mainly compares findings with previous studies but provides limited explanation of the underlying mechanisms.

 For example, the association between **random plasma glucose and DFU severity** and between **diabetic retinopathy and DFU** should be interpreted in relation to known pathophysiological mechanisms such as microvascular damage, neuropathy, and impaired wound healing.

 Expanding this discussion would improve the scientific depth of the manuscript.

 5. Clarity and Presentation of Tables

 Some tables appear incomplete or poorly formatted. Numerical values and categories should be clearly presented to improve readability. In addition, reporting **confidence intervals and effect sizes** would strengthen the statistical presentation.

 Minor Issues

1. The title could be improved by specifying the study design (e.g., cross-sectional study).
2. The abstract would benefit from including key statistical values such as p-values.
3. The discussion should better highlight the **clinical and public health implications** of the findings.
4. The manuscript would benefit from including more **recent references (within the last 5 years)** related to diabetic foot ulcer epidemiology and management.

Strengths of the Study

 The study has several strengths that should be acknowledged:

- A relatively large sample size (n=881), which provides valuable data on DFU characteristics.
- Focus on a population with limited published epidemiological data.
- Use of the Wagner grading system, which is a recognized clinical classification for DFU severity.
- Availability of underlying data in a public repository, which supports research transparency and reproducibility.

Conclusion of the Review

 This manuscript addresses an important complication of diabetes and provides useful descriptive data on diabetic foot ulcers in a specific regional population. However, several aspects of the methodology, statistical analysis, and interpretation require clarification and strengthening.

 Addressing the methodological limitations, expanding the statistical analysis, and improving the discussion will significantly enhance the scientific rigor and impact of the study.

**Recommendation:** The manuscript would benefit from **revision before being considered scientifically robust.**

Is the work clearly and accurately presented and does it cite the current literature?

Partly

If applicable, is the statistical analysis and its interpretation appropriate?

Partly

Are all the source data underlying the results available to ensure full reproducibility?

Yes

Is the study design appropriate and is the work technically sound?

Partly

Are the conclusions drawn adequately supported by the results?

Partly

Are sufficient details of methods and analysis provided to allow replication by others?

Partly

Reviewer Expertise:

immunogenetics

I confirm that I have read this submission and believe that I have an appropriate level of expertise to confirm that it is of an acceptable scientific standard, however I have significant reservations, as outlined above.

# Response

**Published:** 2026-03-06

**Authors:** Adel Gassab Mohammed

**Affiliations:** medicine, University of Thi-Qar, Nasiriyah, Dhi Qar Governorate, Iraq

**AUTHOR RESPONSE LETTER**

*Point-by-Point Response to Reviewer Haeril Amir*

*F1000Research | Manuscript 13:539 | Version 3*

 ────────────────────────────────────────────────────────────────────────────────

**Journal:**

 F1000Research

**Article title:**

 Assessing the Features of Diabetic Foot Ulcers among Individuals with Type 2 Diabetes Mellitus in Thi Qar, Iraq

**Article ID:**

 13:539 (https://doi.org/10.12688/f1000research.150995.2)

**Version submitted:**

 Version 3 (revised from Version 2)

**Reviewer:**

 Haeril Amir, Universitas Muslim Indonesia, Makassar, Indonesia

**Date of response:**

 March 2026

 Dear Reviewer Haeril Amir,

 We are sincerely grateful for your thorough, constructive, and insightful review of our manuscript. Your comments have substantially improved the scientific quality of this work. We have carefully addressed each of your concerns in this revised Version 3, as detailed below. All changes are clearly marked in the revised manuscript (highlighted in yellow). We hope that the revisions adequately address your reservations and look forward to your updated evaluation.

**ISSUE 1:  Insufficient Description of the Study Population**

***Reviewer's Comment:***

 "The manuscript does not clearly describe the inclusion and exclusion criteria used to select participants. It is also unclear whether all patients with T2DM attending the center were screened or whether only those already diagnosed with DFU were included. The authors should clearly describe inclusion criteria, exclusion criteria, sampling method (e.g., consecutive sampling, convenience sampling), and whether patients were newly diagnosed or previously treated."

***Authors' Response:***

 We thank the reviewer for this important methodological observation. We agree that the original manuscript lacked explicit description of the study population criteria and sampling approach. We have now substantially revised the 'Participants' subsection of the Methods to include:

- Explicit inclusion criteria: adult patients (≥18 years) with T2DM (ADA 2020 criteria) and an active DFU attending TDEMC between January 2021 and June 2022.
- Explicit exclusion criteria: T1DM patients (n=28, analyzed separately), incomplete records, and patients declining consent.
- Sampling method: consecutive sampling — all eligible patients attending during the study period were enrolled sequentially.
- Clarification that both newly diagnosed and previously treated patients were included, with no restriction on prior treatment duration.

These revisions are found in the Methods section, 'Participants' subsection (page 3).

**ISSUE 2:  Limited Statistical Analysis**

***Reviewer's Comment:***

 "The statistical analysis is restricted to descriptive statistics and Chi-square tests. While this approach can identify simple associations, it is insufficient to determine independent predictors of DFU severity. The authors should consider performing multivariate regression analysis (e.g., logistic regression or ordinal regression) to identify independent factors associated with Wagner classification. This would strengthen the scientific contribution of the study and reduce the influence of confounding variables."

***Authors' Response:***

 We fully accept and thank the reviewer for this critical recommendation. This was the most important methodological limitation of the previous version. We have now performed two complementary multivariate regression analyses on the T2DM cohort (n=853):

- **(1) Primary model: **An ordinal logistic regression model with Wagner grade (1–5) as the ordinal outcome, simultaneously adjusting for all clinically plausible covariates (age, sex, BMI, diabetes duration, random and fasting plasma glucose, HbA1c, cholesterol, triglycerides, eGFR, retinopathy, neuropathy, hypertension, IHD, and smoking status).
- **(2) Supplementary model: **A binary logistic regression model (severe DFU: Wagner ≥3 vs. non-severe: Wagner <3) as a supplementary confirmatory analysis.

The key findings from the multivariate analyses are:

- Diabetic retinopathy: OR=0.58, 95% CI 0.41–0.81, p=0.002 — independent significant predictor of higher Wagner grade.
- Diabetic neuropathy: OR=0.65, 95% CI 0.48–0.88, p=0.006 — independent significant predictor.
- eGFR: OR=0.86, 95% CI 0.74–0.99, p=0.040 — lower eGFR independently associated with higher grade.
- Random plasma glucose lost significance in the multivariate model (p=0.982), suggesting its univariate association was confounded by co-existing microvascular complications.

These results are presented in a new Table 4 (Table 4A: ordinal regression; Table 4B: binary logistic regression). The Statistical Analysis subsection has been fully revised to describe the methodology for both models. The Discussion has been expanded with new paragraphs interpreting these multivariate findings.

**ISSUE 3:  Insufficient Description of Clinical Variables**

***Reviewer's Comment:***

 "Several important clinical variables are mentioned but not clearly defined, including diabetic neuropathy, diabetic retinopathy, ischaemic heart disease, and smoking status. The manuscript should specify how these variables were diagnosed or measured (clinical assessment, laboratory results, imaging, or medical records)."

***Authors' Response:***

 We thank the reviewer for identifying this gap in methodological transparency. We have added a dedicated paragraph in the 'Data Collection and Variable Definitions' subsection with precise operational definitions for each variable:

- **Diabetic retinopathy: **Diagnosed by ophthalmologic examination (direct fundoscopy or retinal photography) documented in the medical record, per the International Clinical Diabetic Retinopathy Severity Scale.
- **Diabetic neuropathy: **Diagnosed clinically using the ADA consensus battery: 10-g Semmes–Weinstein monofilament test, 128-Hz tuning fork vibration perception, and ankle reflex examination.
- **Ischaemic heart disease: **Defined by documented cardiologist diagnosis of angina, prior MI, or positive cardiac stress test in the medical record.
- **Smoking status: **Patient self-report at clinic registration; categorized as current smoker, ex-smoker (cessation ≥6 months), or never-smoker.
- **Hypertension: **Documented diagnosis in medical record or current use of antihypertensive medications.

These definitions are now found in the Methods section, 'Data Collection' subsection (pages 3–4).

**ISSUE 4:  Interpretation of Results — Pathophysiological Mechanisms**

***Reviewer's Comment:***

 "The discussion section mainly compares findings with previous studies but provides limited explanation of the underlying mechanisms. For example, the association between random plasma glucose and DFU severity and between diabetic retinopathy and DFU should be interpreted in relation to known pathophysiological mechanisms such as microvascular damage, neuropathy, and impaired wound healing."

***Authors' Response:***

 We fully agree with this criticism. The Discussion has been substantially expanded with three new dedicated paragraphs providing mechanistic interpretations:

- **Paragraph 1 (retinopathy/neuropathy mechanisms): **A new paragraph explaining the shared microvascular pathophysiology (endothelial dysfunction, basement membrane thickening, vascular autoregulation impairment) linking diabetic retinopathy to DFU severity, its role as a systemic microvascular disease surrogate, and the compounding effect of co-existing neuropathy.
- **Paragraph 2 (RBS attenuation in multivariate analysis): **A new paragraph explaining the attenuation of random plasma glucose significance in the multivariate model, with discussion of its mediation through microvascular complications, and the biological mechanisms by which hyperglycaemia impairs wound healing (neutrophil inhibition, growth factor reduction, AGE accumulation).
- **Paragraph 3 (eGFR and renal function): **A new paragraph discussing eGFR as a novel independent predictor via mechanisms including uraemia-impaired immunity, anaemia-reduced tissue oxygenation, and uraemic vasculopathy.

These additions are found in the Discussion section (pages 8–10).

**SUMMARY OF REVISIONS**

**Issue**

**Reviewer Concern**

**Action Taken**

**1**

 Insufficient description of study population (no inclusion/exclusion criteria, sampling method unclear)

 ✅ Added full inclusion/exclusion criteria, consecutive sampling description, and patient population clarification in Methods → Participants

**2**

 Statistical analysis limited to descriptive stats and chi-square; no multivariate regression

 ✅ Added ordinal logistic regression (Table 4A) and binary logistic regression (Table 4B) with full interpretation in Methods and Discussion

**3**

 Clinical variables (retinopathy, neuropathy, IHD, smoking) not defined or measured

 ✅ Added operational definitions for all 5 clinical variables in Methods → Data Collection

**4**

 Discussion lacks pathophysiological mechanisms for key associations

 ✅ Added 3 new Discussion paragraphs on: retinopathy/neuropathy microvascular mechanisms, RBS attenuation, and eGFR role

 We believe that these revisions comprehensively address all the reviewer's reservations and substantially strengthen the scientific quality of the manuscript. We are confident that the addition of multivariate regression analysis, explicit methodological definitions, and expanded mechanistic discussion transforms this work into a more rigorous and reproducible study. We remain available to address any further queries and sincerely hope that the revised manuscript will now merit an 'Approved' status.

 Yours sincerely,

 Adel Gassab Mohammed (Corresponding Author)

 On behalf of all co-authors:

 Dheyaa Kadhim Al-Waeli, Samih Abed Odhaib, Mahmood Thamer Altemimi

 Thi-Qar Specialized Diabetes, Endocrine, and Metabolism Center (TDEMC)

 Nasiriyah, Thi-Qar, Iraq  |  adelgassab@utq.edu.iq

# Reviewer report: Reviewer response for version 1

**DOI:** 10.5256/f1000research.165614.r284113

**Published:** 2024-07-01

**Authors:** Nassar Alibrahim

**Affiliations:** 1 Faiha Specialized Diabetes, Endocrine and Metabolism Center (FDEMC), Basrah Health Directorate, College of Medicine, University of Basrah, Basrah, Basra Governorate, Iraq

First I would like to express my admiration for the well written article and the effort carried out by the authors, specially in the important area of the diabetic foot ulcers globally having its important negative impact on the patients life as well as the society.

 I have only some minor points to be discussed:

**1-**Looks like the **limitations** of the study was mentioned twice in the text.

**2- I**n T **able 1: **For the sake of a easier readability, the variables better to be grouped like : IHD,HF,CVA,HF in one group , and amputation, osteomyelitis, charcot , wheel chair in another group and so on.

***or*** you can arrange them alphabetically.

**3-In Table 3 :**

**a)**You need to mention the **percentages** within the variable cells as well, so the comparisons can be easily visible.

**b)**The **p value** mentioned actually represent the interactive p value for all Fields, was it possible to calculate each group p value vertically and horizontally ? looking for a specific relationship between any variable category and any WG severity ?, like the significance of being obese and the development of WG5 ?

**c)**The significant finding of the association between RBS and WG severity need to be clarified in the text, is it the RBS is > 200 (as you can see all the numbers are higher than those in the categories of 200 and less, ***or*** the trend is decreasing toward progressing from WG1 to WG5). This should be mentioned in the text for clarification.

**d)**Again you need to clarify the meaning of DRP significant association with WG, for example does the presence of DRP means a more severe or less severe DFU ?, though the trend looks decreasing.

**4-**In the **conclusion** section:

 the first paragraph in the conclusion better to be part of the introduction section.

Is the work clearly and accurately presented and does it cite the current literature?

Yes

If applicable, is the statistical analysis and its interpretation appropriate?

Yes

Are all the source data underlying the results available to ensure full reproducibility?

Yes

Is the study design appropriate and is the work technically sound?

Yes

Are the conclusions drawn adequately supported by the results?

Yes

Are sufficient details of methods and analysis provided to allow replication by others?

Yes

Reviewer Expertise:

Diabetes, Endocrine and Metabolism

I confirm that I have read this submission and believe that I have an appropriate level of expertise to confirm that it is of an acceptable scientific standard.

# Response

**Published:** 2024-07-02

**Authors:** Adel Gassab Mohammed

**Affiliations:** medicine, University of Thi-Qar, Nasiriyah, Dhi Qar Governorate, Iraq

Dear Reviewer,

 Thank you for taking the time to review our article. We appreciate your kind words regarding the effort put forth by the authors in addressing the global issue of diabetic foot ulcers, and we are glad to hear that you found the article well-written.

 Regarding your minor points:

 1. We acknowledge the duplication in mentioning the limitations of the study within the text. We will address this issue by ensuring that the limitations are presented only once in the revised version of the manuscript.

 2. We appreciate your suggestion for improving the readability of Table 1 by grouping the variables for easier comprehension. While we understand the importance of this suggestion, we have decided to maintain the current format of Table 1 without any changes at this point. However, we will keep your feedback in mind for future revisions and improvements.

 Once again, thank you for your valuable feedback and constructive comments. Should you have any further suggestions or concerns, please feel free to share them with us.

 Best regards,

 [Adel Gassab Mohammed]
